# High sensitivity of a keystone forage fish to elevated CO_2_ and temperature

**DOI:** 10.1093/conphys/coz084

**Published:** 2019-11-21

**Authors:** Christopher S Murray, David Wiley, Hannes Baumann

**Affiliations:** 1 Department of Marine Sciences, University of Connecticut, 1080 Shennecossett Road, Avery Point, CT 06340, USA; 2 National Oceanic and Atmospheric Administration, Stellwagen Bank National Marine Sanctuary, NOAA, 175 Edward Foster Road, Scituate, MA 02066, USA

**Keywords:** Ammodytes dubius, climate change, early life-history, ocean variability hypothesis, ocean acidification, multi-stressor experiment

## Abstract

Sand lances of the genus *Ammodytes* are keystone forage fish in coastal ecosystems across the northern hemisphere. Because they directly support populations of higher trophic organisms such as whales, seabirds or tuna, the current lack of empirical data and, therefore, understanding about the climate sensitivity of sand lances represent a serious knowledge gap. Sand lances could be particularly susceptible to ocean warming and acidification because, in contrast to other tested fish species, they reproduce during boreal winter months, and their offspring develop slowly under relatively low and stable *p*CO_2_ conditions. Over the course of 2 years, we conducted factorial *p*CO_2_ × temperature exposure experiments on offspring of the northern sand lance *Ammodytes dubius*, a key forage species on the northwest Atlantic shelf. Wild, spawning-ripe adults were collected from Stellwagen Bank National Marine Sanctuary (Cape Cod, USA), and fertilized embryos were reared at three *p*CO_2_ conditions (400, 1000 and 2100 μatm) crossed with three temperatures (5, 7 and 10 ˚C). Exposure to future *p*CO_2_ conditions consistently resulted in severely reduced embryo survival. Sensitivity to elevated *p*CO_2_ was highest at 10 ˚C, resulting in up to an 89% reduction in hatching success between control and predicted end-of-century *p*CO_2_ conditions. Moreover, elevated *p*CO_2_ conditions delayed hatching, reduced remaining endogenous energy reserves at hatch and reduced embryonic growth. Our results suggest that the northern sand lance is exceptionally CO_2_-sensitive compared to other fish species. Whether other sand lance species with similar life history characteristics are equally CO_2_-sensitive is currently unknown. But the possibility is a conservation concern, because many boreal shelf ecosystems rely on sand lances and might therefore be more vulnerable to climate change than currently recognized. Our findings indicate that life history, spawning habitat, phenology and developmental rates mediate the divergent early life CO_2_ sensitivities among fish species.

## Teaser

**Lay summary** Sand lances are key groups of forage fishes in northern hemisphere ecosystems, but their sensitivity to elevated *p*CO_2_ and temperature is unknown. Our experiments revealed that embryos of the northern sand lance (*Ammodytes dubius)* are unusually sensitive to near future CO_2_ × temperature conditions, which severely reduced hatching success, hatch size and remaining endogenous reserves.

## Introduction

Forage fish are essential trophic components of all marine ecosystems, because as small, schooling zooplanktivores they channel biological production from plankton to piscivorous fish, mammals and sea birds (Pikitch *et al.*, 2012). Forage fish are also commercially exploited (Tacon and Metian, 2009), and populations under intense industrial fishing pressure have frequently collapsed (Essington *et al.*, 2015; Pinsky *et al.*, 2011). The severe ecosystem consequences of such collapses (Becker and Beissinger, 2006; Furness and Tasker, 2000; Hamre, 1994) underscore the importance of managing forage fish populations with precaution (Pikitch et al., 2012). However, managing forage fish stocks is complicated by their tendency to respond strongly to environmental variability via rapid shifts in distribution or abundance (Alder *et al.*, 2008). Marine climate change will therefore impact forage fish in ways that are complex and still highly uncertain (Shannon *et al.*, 2009). Clearly, sustaining forage fish populations requires a greater understanding how these species will respond to concurrent climate stressors such as ocean warming and acidification.

**Fig. 1:**
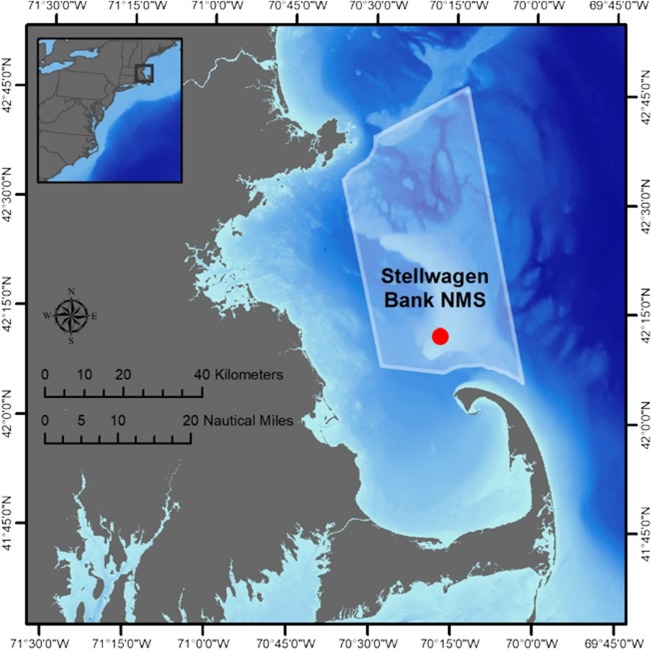
Map of SBNMS and the surrounding region. The red circle denotes the sampling location for spawning-ripe *A. dubius*.

Sand lances in the genus *Ammodytes* are a particularly interesting and important group of forage fish, because its eight species are all locally highly abundant in temperate to polar ecosystems in the northern hemisphere (Orr *et al.*, 2015; Willson *et al.*, 1999). The northern sand lance (*A. dubius*) occurs on sandbanks all along the western North Atlantic shelf, but it is especially important at the Stellwagen Bank National Marine Sanctuary (SBNMS) north of Cape Cod (Fig. 1), where it is the primary forage item for large populations of humpback whales, endangered sea birds, sharks and tunas (Willson et al., 1999) that attract millions of human visitors each year (Wiley *et al.*, 2008). Despite their role in the ecosystem, very little is known about the sensitivity of any sand lance species to the concurrent stressors of marine climate change. Warmer winter temperatures have been shown to depress sand lance recruitment (Arnott and Ruxton, 2002; Lindegren *et al.*, 2018), but detailed experimental work on their temperature sensitivity is scarce. Furthermore, the potential vulnerability of sand lances to future high CO_2_ oceans remains completely unstudied.

As a group, sand lances may be particularly vulnerable to ocean acidification (OA). Like most of its congeners, the northern sand lance spawn during late fall, depositing demersal eggs onto offshore sandbanks where embryos develop slowly under cooling temperatures and relatively low and stable *p*CO_2_ conditions (Salisbury and Jönsson, 2018; Smith *et al.*, 1978). As a consequence, sand lance early life stages likely experience relatively low levels of ambient CO_2_ variability during their development and therefore face weak selection for CO_2_ tolerant phenotypes. This differs from other forage fish species (e.g. Atlantic silverside *Menidia menidia*) that spawn during spring and summer in highly productive, nearshore habitats, where *p*CO_2_ fluctuations often reach maximum levels, which will not occur in the average open ocean for hundreds of years (Baumann and Smith, 2017; Duarte *et al.*, 2013). Organism sensitivities likely decrease with increased environmental variability, as demonstrated, for example, for thermal sensitivities in marine ectotherms (i.e. Climate Variability Hypothesis; Compton *et al.*, 2007; Stevens, 1989; Sunday *et al.*, 2012; Woolsey *et al.*, 2015). A similar framework should also apply to species’ CO_2_ sensitivities [Ocean Variability Hypothesis (OVH); Baumann, 2019] and is consistent with empirical data for some marine invertebrates (Hofmann *et al.*, 2014; Kelly *et al.*, 2013; Vargas *et al.*, 2017) and fish (Hamilton *et al.*, 2017; Jarrold *et al.*, 2017; Jarrold and Munday, 2019).

We experimentally quantified the effects of current to future *p*CO_2_ (400, 1000 and 2100 μatm) and temperature conditions (5, 7 and 10°C) on the northern sand lance offspring. We hypothesized that the slow development of the northern sand lance offspring in relatively CO_2_ stable waters would make this species particularly CO_2_-sensitive. We specifically examined whether exposure to future *p*CO_2_ and temperature conditions affected hatching success, time to hatch, embryonic growth and endogenous energy consumption. Given the similarities in life history between sand lance species, our findings for the northern sand lance may have broad implications for the vulnerability of northern hemisphere marine food webs to the combined stressors of ocean warming and acidification.

## Methods

### Field sampling and fertilization

Spawning-ripe sand lance were collected from SBNMS (42° 9′ 58.26′′ N, 70° 18′ 44.19′′ W; Fig. 1) on 2 December 2016 and 22 November 2017, using a 1.3 × 0.7 m beam trawl (6 mm mesh) towed at 3 knots for 15 min. On deck, adults were checked for ripeness and sorted by sex. For the pilot trial in 2016, experimental embryos were fertilized at sea immediately after spawners were collected (2016: sea, Table S1). For the main experiment in 2017, experimental embryos were produced from two fertilizations (Table S1). The first was completed at sea immediately following the collection of spawners (2017: sea), while the second used adults transported to the Rankin Seawater Facility (University of Connecticut Avery Point) and strip-spawned after being held for 2 days in 400-l circular tanks at 10°C without food (2017: laboratory). In each case, we used at least 10 spawners per sex ranging 14–19 cm in total length (TL; mean, 16 cm; Table S1). Strip-spawning protocols were adapted from early experimental work on the congener *A. americanus* (Smigielski *et al.*, 1984). For each fertilization event, gametes from all spawners were mixed together in plastic spawning trays for 1 hr at 10°C, which was the bottom water temperature at the time of adult collection. Embryos fertilized at sea were maintained in coolers at 10°C during transport to the laboratory. Embryos spawned in the laboratory were treated with 40 g l^−1^ of diatomaceous earth (food grade, Root Naturally®) to better prevent the adhesive eggs from clumping (Smigielski et al., 1984).

### Experimental CO_2_ and temperature conditions

We tested factorial combinations of three *p*CO_2_ × three temperature levels. The target for *p*CO_2_ controls was 400 μatm (~8.15 pH_NIST_), a level characteristic of the average open ocean and of the *A. dubius* spawning habitat in late fall (Salisbury and Jönsson, 2018). As elevated *p*CO_2_ contrasts, we chose 1000 μatm (~7.78 pH) and 2100 μatm (~7.48 pH), which correspond to predicted average ocean *p*CO_2_ levels by the years 2100 and 2300, respectively (Caldeira and Wickett, 2005). The three experimental temperatures were 5, 7 and 10°C, which encompass the current range of decreasing temperature conditions on Stellwagen Bank during the estimated spawning period of northern sand lance in late fall and early winter (Salisbury and Jönsson, 2018; Smith et al., 1978). Given the rapid warming already observed in the Gulf of Maine (Pershing *et al.*, 2015) and potential warming of 2–3°C over the next century (Alexander *et al.*, 2018), the warm 10°C treatment represents a thermal condition likely to become more common during the reproductive period of *A. dubius*.

We developed a LabView (National Instruments®) program to automate *p*CO_2_ manipulations in treatment tanks as described in detail by Murray & Baumann (2018). The pH and dissolved oxygen (DO) conditions of each tank were monitored by a central pH electrode (Hach pHD®, calibrated weekly using 2-point pH_NIST_ references to nearest 0.01) and an optical DO probe (Hach LDO® Model 2). DO levels were maintained at ~ 100% saturation. Temperature was monitored by an embedded sensor in the LDO probe and was controlled in each tank by thermostats (Aqualogic®) that powered in-line chillers (DeltaStar®). Realized treatment *p*CO_2_ conditions were calculated based on measurements of pH, temperature, salinity and total alkalinity (*A*_T_). For measurements of *A*_T_ (μmol kg^−1^), treatment tanks were sampled three times during each experiment by filtering (to 10 μm) treatment seawater into 300-ml borosilicate bottles. Salinity was measured at the time of sampling using a refractometer. Bottles were stored at 3°C and measured for *A*_T_ within two weeks of sampling using an endpoint titration (Mettler Toledo® G20 Potentiometric Titrator). Methodological accuracy (within ±1%) of alkalinity titrations were verified and calibrated using Dr Andrew Dickson’s certified reference material for *A*_T_ in seawater (University of California San Diego, Scripps Institution of Oceanography, Batch Nr. 162 & 164). Unmeasured carbonate parameters were calculated in CO2SYS (V2.1, http://cdiac.ornl.gov/ftp/co2sys) as described by Murray & Baumann (2018). Treatment levels and measurements of carbonate chemistry for this study are reported in Table 1.

**Table 1:** Carbon chemistry and temperature measurements from CO_2_ × temperature factorial experiments (pilot and main experiment) on *A. dubius* offspring

Exp.	Target temp	Temp	Target *p*CO_2_	pH	*p*CO_2_	*A_T_*	*C_T_*	*f*CO_2_	CO_3_^2−^
Pilot	5	5.4 ± 0.1	400	8.08 ± 0.02	446 ± 3	2137 ± 17	2032 ± 17	444 ± 3	23 ± 0.2
	5	5.4 ± 0.1	1000	7.80 ± 0.01	890 ± 3	2132 ± 8	2108 ± 8	886 ± 3	46 ± 0.1
	5	5.3 ± 0.2	2100	7.50 ± 0.03	1828 ± 8	2137 ± 10	2197 ± 10	1821 ± 8	96 ± 0.4
	10	10.0 ± 0.8	400	8.09 ± 0.01	453 ± 4	2143 ± 19	2013 ± 18	451 ± 4	20 ± 0.1
	10	10.3 ± 0.8	1000	7.81 ± 0.01	909 ± 6	2125 ± 16	2082 ± 15	905 ± 6	40 ± 0.3
	10	10.3 ± 0.8	2100	7.51 ± 0.03	1881 ± 2	2131 ± 3	2171 ± 3	1873 ± 2	83 ± 0.1
Main	5	4.9 ± 0.2	400	8.15 ± 0.06	385 ± 11	2198 ± 69	2074 ± 65	384 ± 11	97 ± 4
	5	4.9 ± 0.2	1000	7.72 ± 0.04	1103 ± 49	2175 ± 103	2177 ± 103	1099 ± 49	38 ± 2
	5	4.8 ± 0.3	2100	7.43 ± 0.07	2180 ± 68	2159 ± 76	2246 ± 79	2171 ± 68	19 ± 1
	7	7.1 ± 0.3	400	8.16 ± 0.05	379 ± 9	2177 ± 64	2039 ± 59	377 ± 9	105 ± 4
	7	7.0 ± 0.3	1000	7.74 ± 0.07	1076 ± 29	2185 ± 71	2174 ± 69	1072 ± 28	43 ± 2
	7	7.2 ± 0.3	2100	7.45 ± 0.08	2155 ± 74	2177 ± 82	2250 ± 84	2146 ± 74	22 ± 1
	10	10.0 ± 0.4	400	8.15 ± 0.04	404 ± 9	2219 ± 41	2066 ± 41	402 ± 9	116 ± 1
	10	9.7 ± 0.4	1000	7.76 ± 0.04	1051 ± 10	2180 ± 22	2153 ± 22	1047 ± 10	50 ± 1
	10	9.5 ± 0.4	2100	7.48 ± 0.03	2056 ± 23	2173 ± 34	2228 ± 33	2048 ± 23	26 ± 1

Mean (±SD) pH (NIST) and temperature (°C) are from daily measurements. Mean (±SD) salinity, total alkalinity (*A*_T_; μmol kg^−1^), dissolved inorganic carbon (*C*_T_; μmol kg^−1^), partial pressure and fugacity of CO_2_ (*p*CO_2_; *f*CO; μatm) and carbonate ion concentration (CO_3_^2−^; μmol kg^−1^) quantified from replicated seawater samples. Salinity was measured via refractometer and *A*_T_ from endpoint titrations while *p*CO_2_, *C_T_*, *f*CO_2_ and CO_3_^2−^ were calculated in CO2SYS.

Salinity was 31 psu for all treatments.

### Experimental design

Sand lance embryos and larvae were reared in a purpose-built system consisting of nine recirculating units that each housed up to five replicate rearing containers (20-l polyethylene buckets). Embryos developed in customized baskets (meshed, 2-l polyethylene cups) floating inside each rearing container and received a continuous flow of 4 l h^−1^. All offspring were reared under a light cycle of 11L:13D and a salinity of 31. For the pilot trial, equal amounts of embryos were randomly distributed into replicate rearing containers per *p*CO_2_ treatment (n = 2 for 400; 2100 μatm; n = 1 for 1000 μatm) and temperature (5 and 10°C) within 9 hrs post-fertilization. The exact number of embryos allotted to each rearing basket was confirmed at 130 degree days post-fertilization (dpf) (degree days = rearing temperature * days, ddpf) after examination via dissecting microscope (8× mag). Thereafter, embryo baskets were monitored daily, and hatched larvae were counted and immediately preserved in a 5% formaldehyde/freshwater solution saturated with sodium tetraborate buffer. Standard length at hatch (SL, nearest 0.01 mm) was measured via calibrated microscope images using Image Pro Premier (V9.0, Media Cybernetics®). The pilot trial was terminated after 400 ddpf to fully encapsulate the potential hatching period (40 days at 10°C and 80 days at 5°C).

For the main experiment, equal amounts of embryos (2.5 ml or ~ 3000 embryos) from the fertilization at sea were randomly placed into each of two replicate rearing containers per factorial *p*CO_2_ (400; 1000; 2100 μatm) and temperature combination (5, 7 10°C) within 9 hrs of fertilization. Two days later, 300 embryos from the fertilization in the laboratory were distributed into three additional replicate rearing containers in six factorial *p*CO_2_ (400; 1000; 2100 μatm) and temperature combinations (5 and 10°C) within 2 hrs post-fertilization. Low availability of laboratory fertilized embryos precluded additional replicates for all 7°C *p*CO_2_ treatments (see Fig. S1 for a schematic of the experimental design).

Daily checks for hatchlings started at 100 ddpf, and hatchlings were counted and then preserved. For embryos fertilized at sea, subsamples (n ≥ 12) for morphometric measurements were taken on the first day when 10 or more larvae hatched in a given replicate, whereas all hatchlings from the laboratory fertilization were preserved for morphometric measurements (Table S2). Daily monitoring of 7°C treatments was discontinued after initial subsampling, but daily counts of hatchlings continued for all 5 and 10°C treatments until 400 ddpf.

**Fig. 2:**
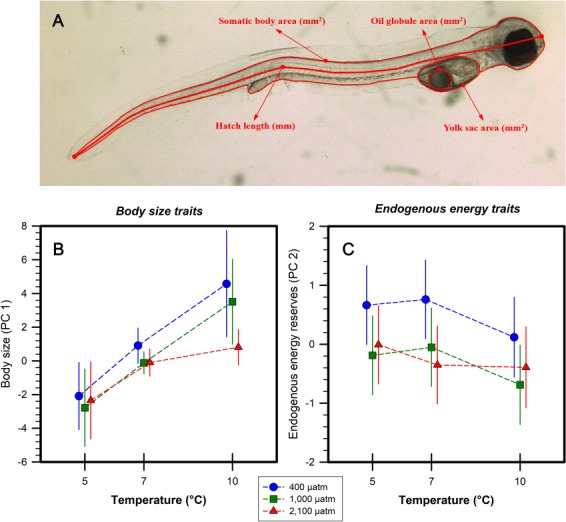
*A. dubius.* (**A**) Schematic of morphometric measurements made on newly hatched larvae from the main experiment. Measurements include hatch length (SL, nearest 0.01 mm), somatic body area (nearest 0.01 mm^2^), yolk sac area (nearest 0.001 mm^2^) and oil globule area (nearest 0.001 mm^2^). (**B, C**) Sea fertilization. Model-adjusted (±95% confidence interval) treatment PC scores of body size traits (B; PC 1; SL and somatic body area) and endogenous energy reserves (C; PC 2; yolk sac and oil globule area) per temperature and *p*CO_2_ treatment (blue circles, 400 μatm; green squares, 1000 μatm; red triangles, 2100 μatm).

### Response traits

Replicate-specific hatching success (%) was calculated for the pilot trial and main experiment as the proportion of hatchlings relative to the number of starting embryos (Pilot: ~ 350; Main_Sea_: 3000 Main_Lab_: 300). Treatment-specific daily hatch frequencies (=daily treatment hatch/total hatch within temperature treatment) and replicate-specific average time to hatch (dpf = summed age of all larvae at hatch/total hatch count) were calculated for the main experiment. Hatching success and frequencies were not calculated for main experiment treatments at 7°C. During the main experiment, subsampled hatchlings were measured for two body size metrics [SL and somatic body area (nearest 0.01 mm^2^)] and two measures of remaining endogenous energy reserves, i.e. yolk sac area (nearest 0.001 mm^2^) and oil globule area (nearest 0.001 mm^2^) as illustrated in Fig. 2A. All experimental procedures complied with the ethical guidelines under Institutional Animal Care and Use Committee protocol #A17–043.

### Statistical analysis

We tested for significant treatment effects on offspring hatching success, age at hatch and growth for the main experiment, while simply reporting the pilot trial data for comparison. Proportional data were logit transformed [log_10_(value/(1—value))] prior to analysis, with zero values replaced with 0.001 (Warton and Hui, 2011). A two-way linear mixed-effect model (LMM) was used to test for significant effects (α < 0.05) of *p*CO_2_, temperature, their interaction (fixed factors) and fertilization event (random factor) on hatching success and time to hatch using the model:

$$\begin{align*}\textrm{Hatching trait} =&\ p\textrm{CO}_2 + \textrm{temperature} + p\textrm{CO}_2\\ &\times \textrm{temperature} + \textrm{fertilization} + \textrm{error}.\end{align*}$$

Statistical testing of morphometric measurements was limited to newly hatched subsamples taken from sea fertilized replicates of the main experiment. Because morphometric traits were intercorrelated, we performed principal component (PC) analyses preceded by evaluating sampling adequacy (Kaiser–Meyer–Olkin measure, >0.5) and Bartlett’s test of sphericity (*P <* 0.001). We extracted two rotated PCs (oblimin procedure) with eigenvalues >1 that explained 43 and 39% of the total variance, respectively (82% cumulatively). Component scores were assigned to each larva. Body size metrics loaded positively on PC1 (>0.89), while endogenous energy metrics loaded positively on PC2 (>0.89). A two-way LMM was used to test for significant effects of *p*CO_2_, temperature, their interaction (fixed factors), age_days_ (covariate) and rearing vessel (random factor) on body size and endogenous energy PC scores using the model:

$$\begin{align*}\textrm{PC} =&\ p\textrm{CO}_2 + \textrm{temperature} + p\textrm{CO}_2\times \textrm{temperature}\\ & + \textrm{rearing vessel (tank)} + \textrm{age} + \textrm{error}.\end{align*}$$

Sample age significantly influenced body size scores (Table 2) and the LMM evaluated treatment effects on scores normalized to a sample age of 29.6 days. Sample age did not significantly affect endogenous energy scores and was not included in the final model. All statistical tests were conducted in SPSS (V20, IBM). For all tests, model residuals were checked for normality by visual inspection of Q-Q plots and for variance homogeneity at each level of fixed and random effects via Levene’s test. Bonferroni corrected post hoc tests were applied for multiple comparisons.

**Table 2:** *A. dubius*. Summary statistics of LMMs evaluating *p*CO_2_, temperature and age effects on morphometric PC scores from the main experiment sea fertilization

PC	Factor	Num. df	Den. df	F	*P*
Body size	*p*CO_2_	2	8.264	6.295	**0.022**
	Temp	2	8.116	10.469	**0.006**
	*p*CO_2_ × temp	4	8.220	3.542	0.059
	Age	1	7.900	9.867	**0.014**
Endogenous energy	*p*CO_2_	2	9.836	7.045	**0.013**
	Temp	1	9.796	2.278	0.154
	*p*CO_2_ × temp	2	9.792	0.070	0.825

Numerator (Num.) and denominator (Den.) degrees of freedom (df) are shown for each factor.

Significant effects are denoted in bold.

### Results

#### Hatching success

**Fig. 3:**
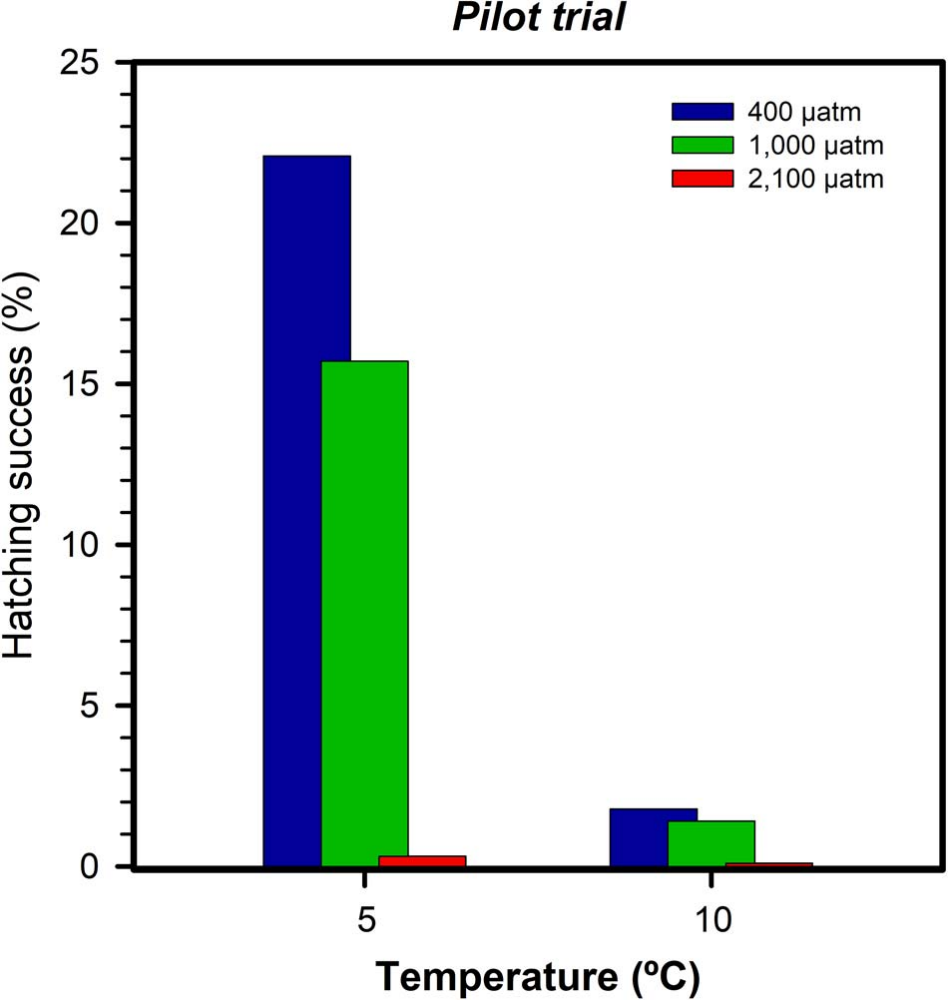
*A. dubius.* Mean embryo survival (%) of offspring reared at 400 μatm (blue, left bar), 1000 μatm (green, central bar) and 2100 μatm *p*CO_2_ (red, right bar) and two temperatures from the pilot trial.

Across years, we found a consistent reduction in hatching success with increasing *p*CO_2_ and temperature conditions. In the pilot trial, hatching success at 5°C was 23% at 400 μatm, 16% at 1000 μatm and 1% under 2100 μatm. At 10°C, hatching success was 2%, 1% and 0% at 400, 1000 and 2100 μatm *p*CO_2_, respectively (Fig. 3).

**Fig. 4:**
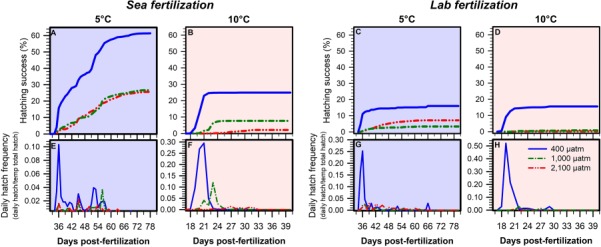
*A. dubius.* Hatching timelines from each fertilization event (2017: sea; 2017: laboratory) tested during the main experiment. Embryos were reared at 5°C (blue background) and 10°C (red background) crossed with three pCO_2_ levels: 400 μatm (blue lines), 1000 μatm (green lines) and 2100 μatm (red lines). Traits presented are hatching success (**A–D**, %) and daily hatch frequencies (**E–H**, daily hatch/total final hatch within temperature treatment).

During the main experiment, mean hatching success varied between and within treatments (Table S3) but was significantly affected by a *p*CO_2_ × temperature interaction (LMM, *P* = 0.001; Table 3, Fig. 4A–D). We found that the warm rearing temperature exacerbated the negative effect of elevated *p*CO_2_ on hatching success. Within the 5°C treatments, mean hatching success under both elevated *p*CO_2_ conditions declined by 62% relative to embryos reared under 400 μatm (Bonferroni, *P* < 0.01). At 10°C, the percent reduction in hatching success under elevated *p*CO_2_ conditions increased to 89% (Bonferroni, *P* < 0.001). For offspring reared under 400 μatm *p*CO_2_, hatching did not vary between temperature treatments, but both elevated *p*CO_2_ treatments significantly reduced hatching success at 10°C relative to 5°C (Bonferroni, *P* < 0.001). Within temperature treatments, hatching rates did not vary between 1000 and 2100 μatm *p*CO_2_. Overall, hatching success was higher in embryos fertilized at sea (5–62% across treatments) compared to embryos fertilized in the laboratory (0–16% across treatments; Fig. 4, Table S3).

**Table 3:** *A. dubius.* Summary statistics from a LMMs testing *p*CO_2_ × temperature effects on hatching success (logit transformed) and age at hatch from the main experiment

Trait	Factor	Num. df	Den. df	F	*P*
Hatching success	*p*CO_2_	2	23	51.942	**<0.001**
	Temp	1	23	73.835	**<0.001**
	*p*CO_2_ × temp	2	23	9.964	**0.001**
Age at hatch	*p*CO_2_	2	22	7.553	**0.004**
	Temp	1	22	388.048	**<0.001**
	*p*CO_2_ × temp	2	22	0.086	0.918

Numerator (Num.) and denominator (Den.) degrees of freedom (df) are shown for each factor.

Significant effects are denoted in bold.

The cold rearing temperature and exposure to elevated *p*CO_2_ significantly increased the average time to hatch, but no interaction was found (LMM, *P* < 0.005; Table 3, Table S3). Embryos reared at 400 μatm *p*CO_2_ hatched in one major peak shortly after first hatch, whereas embryos developing at 1000 and 2100 μatm hatched gradually over a protracted period of time (Fig. 4E–H). This was most pronounced at 10°C, where 99% of embryos at 400 μatm emerged within the first 3 days of hatching compared to only 5% of embryos in 2100 μatm *p*CO_2_ treatment. In the latter, it took 8–12 days for the majority of hatchlings to emerge (Fig. 4F).

### Morphometrics

Treatment means (±SD) for all morphometric measurements can be found in Table S2. An LMM found a significant effect of *p*CO_2_ on body size scores (*P* = 0.022; Table 2, Fig. 2B). Larvae reared under 2100 μatm were significantly smaller at hatch than those exposed to 400 μatm *p*CO_2_ (Bonferroni, *P* = 0.022). Rearing temperature had a significant influence on hatchling body size (LMM, *P* = 0.006; Table 2, Fig. 2B). Offspring reared at 10°C were significantly larger than samples from 5 and 7°C (Bonferroni, *P* < 0.05). A *p*CO_2_ × temperature interaction was not detected (Table 2).

Endogenous energy PC scores were significantly influenced by *p*CO_2_ level (LMM, *P* = 0.013; Table 2, Fig. 2C). Offspring reared under 400 μatm had significantly more endogenous energy reserves at hatch compared to larvae from 1000 and 2100 μatm *p*CO_2_ (Bonferroni, *P* < 0.05). Temperature did not independently affect endogenous energy size, nor did it influence the effect of *p*CO_2_ (Table 2).

## Discussion

Sand lances represent a group of globally important forage fishes, but their vulnerability to marine climate change remains understudied. We conducted the first comprehensive evaluation of early life CO_2_ × temperature sensitivities in a sand lance species (northern sand lance, *A. dubius*) and found that exposure to future *p*CO_2_ and temperature conditions severely reduced hatching success. The lethality of elevated *p*CO_2_ increased with increasing temperature. To date, lethal effects of 1000–2100 μatm *p*CO_2_ in fish early life stages have been documented in some, but not most species (Cattano *et al.*, 2018). Notable examples include reductions in survival of up to 74% in inland silversides (*Menidia beryllina)* (Baumann *et al.*, 2012; Gobler *et al.*, 2018), 48% in summer flounder (*Paralichthys dentatus)* (Chambers *et al.*, 2014) and 47% in Atlantic cod (*Gadus morhua*) (Dahlke *et al.*, 2017). In *A. dubius*, the reductions in embryo survival ranged from 60 to 89% under 1000–2100 μatm *p*CO_2_ (69% on average) relative to contemporary *p*CO_2_ conditions, hence making it one of the most CO_2_ sensitive fish species documented to date (Cattano *et al.,* 2018).

In addition, exposure to acidified conditions reduced the remaining endogenous reserves of yolk-sac hatchlings. Exposure to elevated *p*CO_2_ may have increased energetic demands through increased rates of ion regulation and protein synthesis and turnover (Melzner *et al.*, 2009; Pan *et al.*, 2015). Embryos exposed to 2100 μatm *p*CO_2_ were also significantly smaller at hatch, suggesting that yolk utilization had already been maximized, and further CO_2_ acclimation required metabolic tradeoffs prioritizing energy for homeostasis over somatic growth (Wieser and Krumschnabel, 2001). These effects would likely be detrimental to *A. dubius* larvae in the wild, because smaller larvae with less endogenous energy generally experience higher cumulative mortality rates (Miller *et al.*, 1988). The observed delay in bulk hatching under acidified conditions may further compound these effects. A CO_2_-induced decoupling of embryonic duration with temperature and other drivers of phenology means that the timing of bulk hatch could be offset from optimal resources and predation pressure (Cushing, 1990).

We found that the warm rearing temperature compounded the negative effects of elevated CO_2_ on survival, which is consistent with a growing number of studies reporting negative synergistic effects of elevated *p*CO_2_ and sub-optimal temperatures in fish early life stages (Dahlke et al., 2017; Davis *et al.*, 2018; Flynn *et al.*, 2015; Gobler et al., 2018; Pimentel *et al.*, 2014). Furthermore, the apparent high sensitivity of *A. dubius* embryo survival to elevated *p*CO_2_, and temperature is consistent with findings across other marine taxa (Przeslawski *et al.*, 2015). However, not all fish early life stages have demonstrated synergistic CO_2_ × temperature effects (Lefevre, 2016; Murray and Baumann, 2018); hence, a more detailed mechanistic understanding of CO_2_ × temperature effects in marine fish is required (Jutfelt *et al.*, 2018; Pörtner *et al.*, 2017). Nevertheless, the negative synergistic effect on hatching success is particularly concerning for a species that spawns throughout the Gulf of Maine, one of the fastest warming regions of the ocean (Alexander et al., 2018; Pershing et al., 2015). Warming temperatures have already been linked to depressed sand lance recruitment in the North Sea (Arnott and Ruxton, 2002; Lindegren et al., 2018). Further investigation into the underlying mechanisms of CO_2_ × temperature sensitivity in sand lance are urgently needed as rapid warming in the northwest Atlantic may already be affecting sand lance early life stages.

What exactly caused the *p*CO_2_ lethality in *A. dubius* embryos is unknown. Mortality could have been due to uncompensated acidosis (Kikkawa *et al.*, 2004) impairing pH-sensitive vital processes (Pörtner *et al.*, 2005). Intriguingly, post-experiment observations of rearing baskets showed that dead embryos from acidified treatments appeared pigmented and near full development, but were seemingly unable to hatch. Thus, an alternative explanation could be that elevated *p*CO_2_ disrupted the hatching process itself. This is supported by our observation of prolonged, intermittent hatching under acidified conditions. Hatching in fish is largely dependent on the activity of hatching enzymes, which are pH sensitive and generally perform best in more alkaline conditions (Korwin-Kossakowski, 2012). Therefore, high *p*CO_2_ levels may acidify the perivitelline fluid thereby reducing the proteolytic properties of hatching enzymes and leading to delayed or unsuccessful hatching (Havas and Rosseland, 1995). Hatching enzyme expression or activity has not yet been evaluated in the context of OA, but potentially represents a critical knowledge gap in marine fishes.

During the main experiment of this study, hatching success varied substantially between fertilization events. Relative to the adults spawned in the laboratory, the adults that were strip-spawned at sea produced four-fold more experimental embryos per female, and when averaged across treatments their offspring had better hatching success. This may indicate that the stress of transport combined with a two-day holding period reduced the reproductive quality of the laboratory-spawned adults. Embryos of poor quality likely experience elevated rates of mortality during development (Bobe and Labbé, 2010) and may be more sensitive to abiotic stressors like acidification (Baumann *et al.*, 2018). Therefore, we cannot fully discount that reduced egg quality influenced the effects of elevated *p*CO_2_ in laboratory-fertilized offspring. Low-hatching success of laboratory-fertilized embryos could also have been due to poor fertilization rates. However, fertilization success is not a good proxy for later developmental success in fish early life-stages (Shields *et al.*, 1997) and thus would not influence embryonic *p*CO_2_ sensitivities. Nevertheless, research efforts devoted to improving the spawning and rearing protocols of non-model species like sand lance will improve estimates of their potential climate sensitivity.

**Fig. 5:**
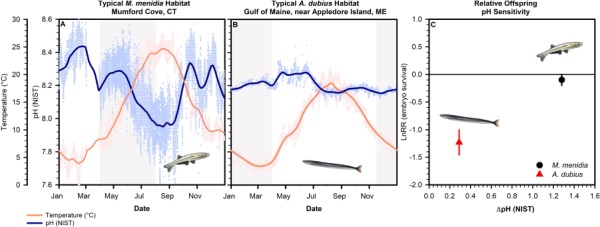
Seasonal pH (NIST, blue) and temperature (°C, red) variability from spawning habitats of *M. menidia* (**A**; Mumford Cove, CT; 41° 19′ 25′′ N 72° 01′ 07″ W; Baumann, 2016) and *A. dubius* (**B**; Coastal Western Gulf of Maine Mooring; 43° 1′ 12′′ N, 70° 32′ 24′′ W; Sutton et al., 2014). The Gulf of Maine mooring is located ~ 100 km north of the sand lance collection site (~10 km offshore, 65 m depth). Faded circles, individual measurements; solid lines, smoothed data; shading, seasonal window of early life stages. (**C**) pH variability vs. pH/CO_2_ sensitivity in *M. menidia* vs. *A. dubius*; average CO_2_ effect (~2000 μatm) on embryo survival (LnRR±95% CI) plotted against the average pH range experienced during spawning season (ΔpH). Data for *M. mendia* (red circle) was taken from Baumann et al. (2018) . In both species, CO_2_-driven reductions in embryo survival (negative LnRRs) are considered significant because 95% confidence intervals do not include 0 (Hedges *et al.*, 1999).

With less than 0.5% of all marine fish species tested, CO_2_ effects appear to be highly species- or even population specific (Cattano et al., 2018), which still precludes generalizations for extrapolating findings to untested taxa. One promising hypothesis is that the CO_2_ tolerance of species and populations increases with the level of *p*CO_2_ variability experienced in their natural habitats (OVH; Baumann, 2019). Consider the well-studied Atlantic silverside (*M. menidia*), another coastal forage fish that spawns in nearshore, sub-tropical to temperate habitats during spring and summer (Conover and Kynard, 1984). These habitats typically undergo seasonal, metabolic acidification with diel pH fluctuations exceeding 0.60 units (7.43–8.10 pH; Fig. 5A). Years of serial experimentation on the species revealed that wild silverside embryos are weakly affected by elevated *p*CO_2_ early in the season, but become progressively more CO_2_ tolerant (Baumann et al., 2018) until by early summer they are unaffected by even extreme *p*CO_2_ conditions (>4000 μatm) (Murray and Baumann, 2018).

Mechanisms that rapidly enhance offspring CO_2_ tolerance are likely adaptive for nearshore species that spawn embryos into highly variable environments (Hoffmann and Hercus, 2000) and therefore appear tolerant of future *p*CO_2_ conditions in experimental settings (Hamilton et al., 2017; Hofmann et al., 2014; Maas *et al.*, 2012; Vargas et al., 2017). In the Gulf of Maine, *A. dubius* spawn in late fall and deposit embryos onto offshore sandbanks, where *p*CO_2_ conditions generally resemble stable surface ocean levels (Salisbury and Jönsson, 2018). Since pH values never fall below 8.00 (Fig. 5B), *A. dubius* offspring may face little selection for CO_2_ tolerant phenotypes, assuming that such tolerance is metabolically costly and thus not maintained if not needed (Sunday *et al.*, 2014). As a result, the sensitivity of *A. dubius* embryos to 2100 μatm *p*CO_2_ appears to be one order of magnitude higher than in silversides (Fig. 5C).

Some empirical assessments of fish early life CO_2_ sensitivities appear to contradict the OVH, e.g. in yellowtail kingfish (*Seriola lalandi*), which spawns in subtropical, pelagic waters with little *p*CO_2_ variability but produces largely CO_2_ resilient offspring (Munday *et al.*, 2016; Watson *et al.*, 2018). However, offspring of *S. lalandi* and other tropical piscivores develop very rapidly, and larvae become active predators within a week of fertilization (Chen *et al.*, 2006). The early onset of rapid growth and mobility likely requires advanced acid-base regulatory capabilities to develop early in ontogeny (Melzner et al., 2009). By contrast, offspring of species like *A. dubius* with protracted early development may have a lower buffering capacity for metabolic CO_2_ and are consequently more sensitive to acidification (Baumann, 2019; Melzner et al., 2009). Variability in stage duration may thus explain why CO_2_ effects observed in tropical species are generally sub-lethal (Bignami *et al.*, 2014; Munday *et al.*, 2009; Welch and Munday, 2016, but see Frommel *et al.*, 2016), whereas examples of direct CO_2_-induced mortality are mostly represented by higher latitude species (Chambers et al., 2014; Dahlke et al., 2017; Flynn et al., 2015; Pimentel et al., 2014).

The OVH framework incorporates natural *p*CO_2_ variability and stage duration and may thus prove useful for predicting early life climate sensitivities and for refocusing research efforts on species likely affected first by OA. The present study provides evidence to support this framework. However, slow-developing fish that spawn in relatively stable CO_2_ environments are severely understudied with respect to CO_2_ × temperature impacts, likely because working with offshore and winter spawners from temperate to polar regions is logistically challenging. To date, experimenters often choose study species based on availability or familiarity. However, robust evaluations of OA × warming sensitivity in the ocean will only be achieved by choosing study species based on concrete theoretical frameworks rather than logistical convenience.

Our discovery that wild embryos of *A. dubius* are exceptionally sensitive to near-future *p*CO_2_ and temperature conditions has direct conservation implications for marine ecosystems. First, many sand lance species share reproductive and early life history characteristics with *A. dubius*, hence there is the potential that other sand lances are equally sensitive to warming and acidifying oceans. Given the central importance of these forage fishes, their elevated climate sensitivity may pose a greater risk to the productivity and resiliency of coastal shelf ecosystems across the northern hemisphere than currently acknowledged. If so, iconic higher trophic predators reliant on sand lance may be challenged. Second, this also means that existing plans for large-scale exploitation of sand lance populations by industrial fisheries should be viewed more skeptically or at least should be proceed with extra precaution due to their heightened sensitivity to marine climate change (Lindegren et al., 2018). Our study thus provides a clear mandate for follow-up research to understand the mechanisms and prevalence of near-future *p*CO_2_ lethality in this ecologically important group of forage fish.

## Supplementary Material

CONPHYS-2019-086R1_Murray_etal_Electronic_supplementary_material_coz084
