# Supplementary material for: High sensitivity of a keystone forage fish to elevated CO2 and temperature
Source: Conserv Physiol. 2019 Nov 21;7(1):coz084. doi: 10.1093/conphys/coz084 (PMC6868386; doi:10.1093/conphys/coz084)
Supplement: CONPHYS-2019-086R1_Murray_etal_Electronic_supplementary_material_coz084 [file conphys-2019-086r1_murray_etal_electronic_supplementary_material_coz084.docx]

***Electronic supplementary material***

**High sensitivity of a keystone forage fish to elevated CO_2_ and temperature**

**Tables**

**Table S1:** Spawning ripe *A. dubius* were collected from Stellwagen Bank National Marine Sanctuary to fertilize embryos used in factorial CO_2_ × temperature experiments. Adult lengths are shown as mean total lengths (TL, cm) ± standard deviation.

| **Fertilization** | **Collection date** | **Fertilization date** | **# Female spawners** | **Female TL** | **# Male spawners** | **Male TL** |
| --- | --- | --- | --- | --- | --- | --- |
| 2016: sea | 12/02/2016 | 12/2/2016 | 13 | 16.3±1.9 | 10 | 16.2±2.2 |
| 2017: sea | 11/22/2017 | 11/22/2017 | 25 | 16.1±1.0 | 27 | 16.0±1.3 |
| 2017: lab | 11/22/2017 | 11/24/2017 | 14 | 15.9±1.1 | 26 | 15.7±1.0 |

**Table S2:** Ages and sample-sizes (N) of newly-hatched *A. dubius* larvae from pilot and main experiment fertilizations. Morphometric measurements shown as treatment mean (± s.d.) hatch length (HL, mm), somatic body area (SA, mm^2^), yolk sac area (YSA, mm^2^), and oil globule area (OGA, mm^2^). Sample age ranges are shown as days post-fertilization (dpf).

| **Fert.** | **Temp (°C)** | ***p*CO_2_ (µatm)** | **Age (dpf)** | **N** | **HL (mm)** | **SA (mm^2^)** | **YSA (mm^2^)** | **OGA (mm^2^)** |
| --- | --- | --- | --- | --- | --- | --- | --- | --- |
| 2016: sea | 5 | 400 | 42 | 10 | 5.84 ± 0.32 | - | - | - |
|  |  | 1,000 | 45 | 20 | 5.78 ± 0.57 | - | - | - |
| 2017:sea | 5 | 400 | 35 | 38 | 5.47 ± 0.30 | 1.26 ± 0.16 | 0.167 ± 0.041 | 0.050 ± 0.017 |
|  |  | 1,000 | 36 | 39 | 5.42 ± 0.34 | 1.22 ± 0.12 | 0.124 ± 0.038 | 0.041 ± 0.014 |
|  |  | 2,100 | 36 | 41 | 5.59 ± 0.16 | 1.25 ± 0.08 | 0.140 ± 0.025 | 0.040 ± 0.009 |
|  | 7 | 400 | 27 | 40 | 5.24 ± 0.23 | 1.21 ± 0.10 | 0.184 ± 0.036 | 0.047 ± 0.014 |
|  |  | 1,000 | 29 | 40 | 5.22 ± 0.20 | 1.18 ± 0.10 | 0.148 ± 0.029 | 0.038 ± 0.010 |
|  |  | 2,100 | 28 | 48 | 5.12 ± 0.22 | 1.13 ± 0.14 | 0.139 ± 0.049 | 0.033 ± 0.012 |
|  | 10 | 400 | 20 | 28 | 5.41 ± 0.20 | 1.28 ± 0.11 | 0.159 ± 0.028 | 0.039 ± 0.010 |
|  |  | 1,000 | 22 | 32 | 5.34 ± 0.23 | 1.25 ± 0.12 | 0.114 ± 0.040 | 0.033 ± 0.012 |
|  |  | 2,100 | 27 | 24 | 5.23 ± 0.29 | 1.18 ± 0.13 | 0.123 ± 0.064 | 0.037 ± 0.016 |
| 2017:lab | 5 | 400 | 35-66 | 128 | 5.51 ± 0.34 | 1.30 ± 0.17 | 0.144 ± 0.057 | 0.041 ± 0.019 |
|  |  | 1,000 | 35-62 | 28 | 5.40 ± 0.38 | 1.27 ± 0.13 | 0.108 ± 0.056 | 0.035 ± 0.025 |
|  |  | 2,100 | 35-64 | 62 | 5.33 ± 0.36 | 1.25 ± 0.14 | 0.107 ± 0.046 | 0.028 ± 0.017 |
|  | 10 | 400 | 18-25 | 121 | 5.21 ± 0.07 | 1.27 ± 0.04 | 0.176 ± 0.044 | 0.059 ± 0.010 |
|  |  | 1,000 | 22-27 | 6 | 5.22 ± 0.35 | 1.25 ± 0.07 | 0.185 ± 0.047 | 0.064 ± 0.024 |
|  |  | 2,100 | 21-24 | 2 | 5.60 ± 0.34 | 1.34 ± 0.13 | 0.148 ± 0.050 | 0.064 ± 0.021 |

**Table S3:** *A. dubius*. Treatment mean hatching success (% ± s.d.) and time to hatch (dpf ± s.d) of offspring from the 2017 sea and lab fertilizations.

| **Fert.** | **Temp (°C)** | ***p*CO_2_ (µatm)** | **Hatching success (% ± s.d.)** | **Age at hatch (dpf ± s.d.)** |
| --- | --- | --- | --- | --- |
| 2017: sea | 5 | 400 | 62 ± 9 | 46 ± 1 |
|  |  | 1,000 | 26 ± 18 | 49 ± 7 |
|  |  | 2,100 | 25 ± 5 | 51 ± 5 |
|  | 10 | 400 | 23 ±5 | 21 ± 1 |
|  |  | 1,000 | 8 ± 5 | 23 ± 1 |
|  |  | 2,100 | 2 ± 1 | 28 ± 1 |
| 2017: lab | 5 | 400 | 16 ± 3 | 39 ± 1 |
|  |  | 1,000 | 3 ± 1 | 42 ± 4 |
|  |  | 2,100 | 7 ± 2 | 45 ± 2 |
|  | 10 | 400 | 16 ± 2 | 20 ± 1 |
|  |  | 1,000 | 1 ± 1 | 23 ± 1 |
|  |  | 2,100 | 1 ± 1 | 23 ± 2 |

**Figures**

**
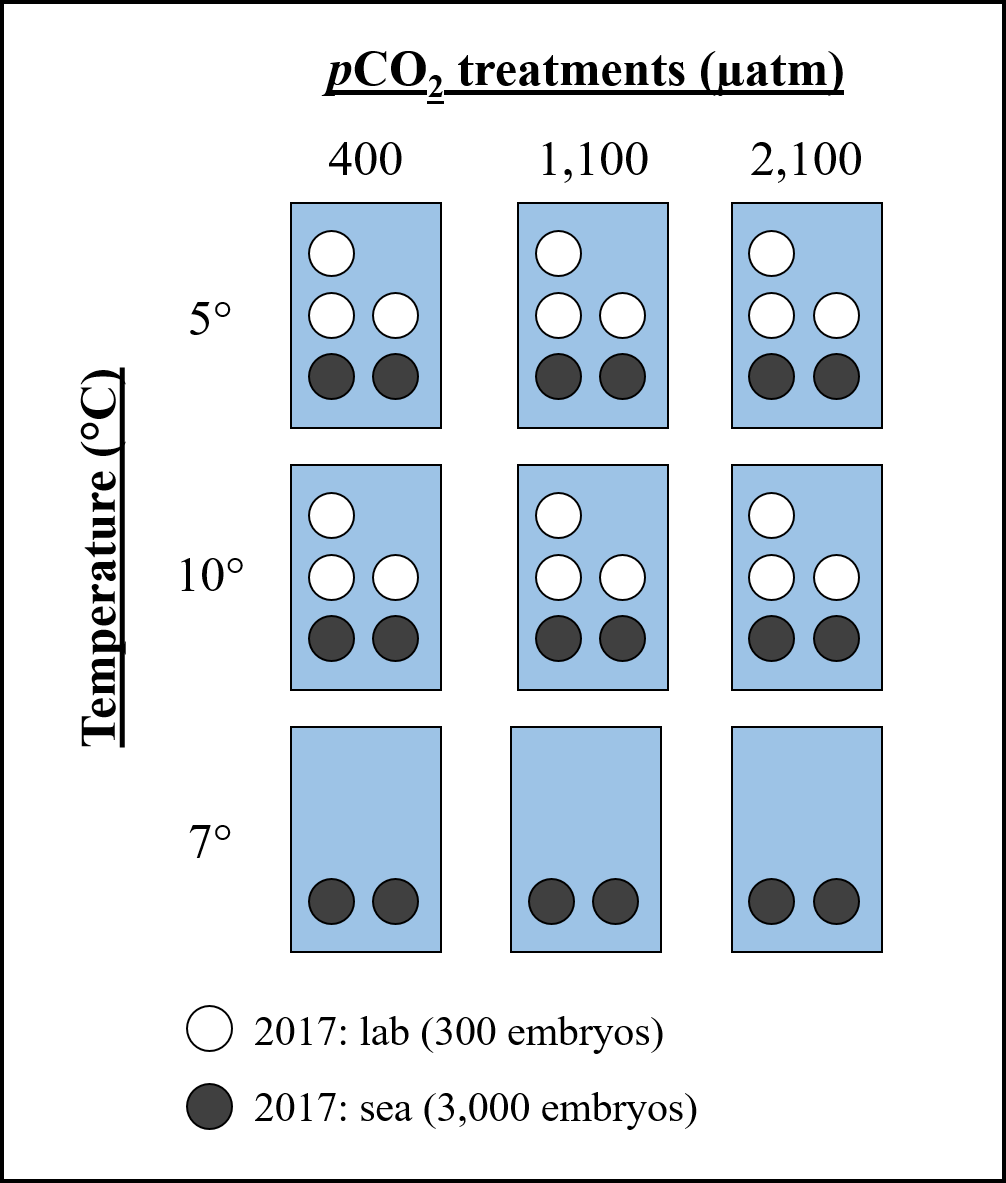
**

**Fig. S1:** Experimental design for the main experiment conducted on *A. dubius* in 2017. The schematic displace the factorial *p*CO_2 ×_ temperature treatment combinations, the distribution of eggs from each fertilization event to replicate rearing vessels, and the number of replicates for each fertilization per treatment combination. White circles represent replicates with eggs from the 2017: lab fertilization (300 per replicate) and black circles are replicates with eggs from the 2017: sea fertilization (3,000 per replicate).
